# Publisher Correction: Multiple fermion scattering in the weakly coupled spin-chain compound YbAlO_3_

**DOI:** 10.1038/s41467-021-24744-y

**Published:** 2021-07-15

**Authors:** S. E. Nikitin, S. Nishimoto, Y. Fan, J. Wu, L. S. Wu, A. S. Sukhanov, M. Brando, N. S. Pavlovskii, J. Xu, L. Vasylechko, R. Yu, A. Podlesnyak

**Affiliations:** 1 Max Planck Institute for Chemical Physics of Solids, Dresden, Germany; 2 Institut für Festkörper- und Materialphysik, Technische Universität Dresden, Dresden, Germany; 3 Department of Physics, Technical University Dresden, Dresden, Germany; 4 Institute for Theoretical Solid State Physics, IFW Dresden, Dresden, Germany; 5 Department of Physics and Beijing Key Laboratory of Opto-Electronic Functional Materials and Micro-Nano Devices, Renmin University of China, Beijing, China; 6 Beijing National Laboratory for Condensed Matter Physics and Institute of Physics, Chinese Academy of Sciences, Beijing, China; 7 Tsung-Dao Lee Institute and School of Physics and Astronomy, Shanghai Jiao Tong University, Shanghai, China; 8 Neutron Scattering Division, Oak Ridge National Laboratory, Oak Ridge, TN USA; 9 Department of Physics, Southern University of Science and Technology, Shenzhen, China; 10 Kirensky Institute of Physics, Federal Research Center, Krasnoyarsk, Russia; 11 Helmholtz-Zentrum Berlin für Materialien und Energie, Berlin, Germany; 12 Heinz Maier-Leibnitz Zentrum, Technische Universität München, Garching, Germany; 13 Present Address: Lviv Polytechnic National University, Lviv, Ukraine; 14 Present Address: Paul Scherrer Institute, Villigen PSI, Villigen, Switzerland

**Keywords:** Electronic properties and materials, Magnetic properties and materials

Correction to: *Nature Communications* 10.1038/s41467-021-23585-z, published online 14 June 2021.

The original version of this Article contained an error in ref. 20, which was incorrectly given with the wrong title as: “Agrapidis, C. E., van den Brink, J. & Nishimoto, S. Ground state and low-energy excitations of the Kitaev-Heisenberg two-leg ladder. *Phys. Rev. B* **99**, 224423 (2019)”. The correct form of ref. 20 is: “Agrapidis, C. E., van den Brink, J. & Nishimoto, S. Field-induced incommensurate ordering in Heisenberg chains coupled by Ising interaction: model for ytterbium aluminum perovskite YbAlO3. *Phys. Rev. B* **99**, 224423 (2019)”.

This has been corrected in the PDF and HTML versions of the Article.

