# Supplementary material for: Multiple fermion scattering in the weakly coupled spin-chain compound YbAlO3
Source: Nat Commun. 2021 Jun 14;12:3599. doi: 10.1038/s41467-021-23585-z (PMC8203633; doi:10.1038/s41467-021-23585-z)
Supplement: Supplementary file 1 — Supplementary Information [file 41467_2021_23585_MOESM1_ESM.pdf]

SUPPLEMENTARY INFORMATION: Multiple fermion scattering in the weakly coupled spin-chain compound $YbAlO_3$

S. E. Nikitin,[1,2,*] S. Nishimoto,[3,4] Y. Fan,[5,†] J. Wu,[6] L. S. Wu,[7,8] A. S. Sukhanov,[1,2] M. Brando,[1] N. S. Pavlovskii,[9] J. Xu,[10,‡] L. Vasylechko,[11] R. Yu,[5] and A. Podlesnyak[7]

[1]*Max Planck Institute for Chemical Physics of Solids, Nöthnitzer Str. 40, D-01187 Dresden, Germany*

[2]*Institut für Festkörper- und Materialphysik, Technische Universität Dresden, D-01069 Dresden, Germany*

[3]*Department of Physics, Technical University Dresden, 01069 Dresden, Germany*

[4]*Institute for Theoretical Solid State Physics, IFW Dresden, 01069 Dresden, Germany*

[5]*Department of Physics and Beijing Key Laboratory of Opto-Electronic Functional Materials and Micro-Nano Devices, Renmin University of China, Beijing 100872, China*

[6]*Tsung-Dao Lee Institute and School of Physics and Astronomy, Shanghai Jiao Tong University, Shanghai 200240, China*

[7]*Neutron Scattering Division, Oak Ridge National Laboratory, Oak Ridge, Tennessee 37831, USA*

[8]*Department of Physics, Southern University of Science and Technology, Shenzhen 518055, China*

[9]*Kirensky Institute of Physics, Federal Research Center, Krasnoyarsk 660036, Russia*

[10]*Helmholtz-Zentrum Berlin für Materialien und Energie, Hahn-Meitner-Platz 1, D-14109 Berlin, Germany*

[11]*Lviv Polytechnic National University, 79013 Lviv, Ukraine*

[*] Present address: Paul Scherrer Institute, Villigen PSI CH-5232, Switzerland;

Corresponding authors: stanislav.nikitin@psi.ch, rong.yu@ruc.edu.cn

[†] Present address: Beijing National Laboratory for Condensed Matter Physics and Institute of Physics, Chinese Academy of Sciences, Beijing, 100190, China

[‡] Present address: Heinz Maier-Leibnitz Zentrum, Technische Universität München, 85748 Garching, Germany

S1. Sample twin structure and origin of the second splitting

$YbAlO_3$ crystallizes in a distorted pervoskite structure with the symmetry group $Pbnm$. The room-temperature lattice parameters are found to be $a = 5.1261(1)$ Å, $b = 5.3314(1)$ Å and $c = 7.3132(2)$ Å, and the a and b values are rather close [1]. Here we provide magnetization and diffraction data to prove that a small part our sample contains twinned domains, which are rotated by 90° around the [001] direction with respect to the primary crystalline axes, similar to the isostructural orthorhombic perovskites [2–4]. It causes the second splitting of the (0 0 1) magnetic reflection in Figs. 2 and 3 in the main text and strongly affects the intensity of the (0 0 1) reflection.

1. *Single-ion anisotropy of Yb ions*

First of all we discuss the magnetic anisotropy of $YbAlO_3$. Point-charge model calculations and magnetization measurements have shown that the Yb moments in $YbAlO_3$ have strong single-ion anisotropy [5, 6] with $g_z \gg g_{xy}$. The Yb moments are confined within the ab-plane and the quantization axis z is canted by an angle $\alpha = \pm 23.5°$ from the [100] direction. Therefore, the magnetic response of $YbAlO_3$ under the influence of a magnetic field applied along the a and b axes is qualitatively similar, because it is caused by the projection of the magnetic field on the easy z-axis of the Yb ions: $M_a = \mu_B g_z \cdot B\cos(\alpha); M_b = \mu_B g_z \cdot B\sin(\alpha)$.

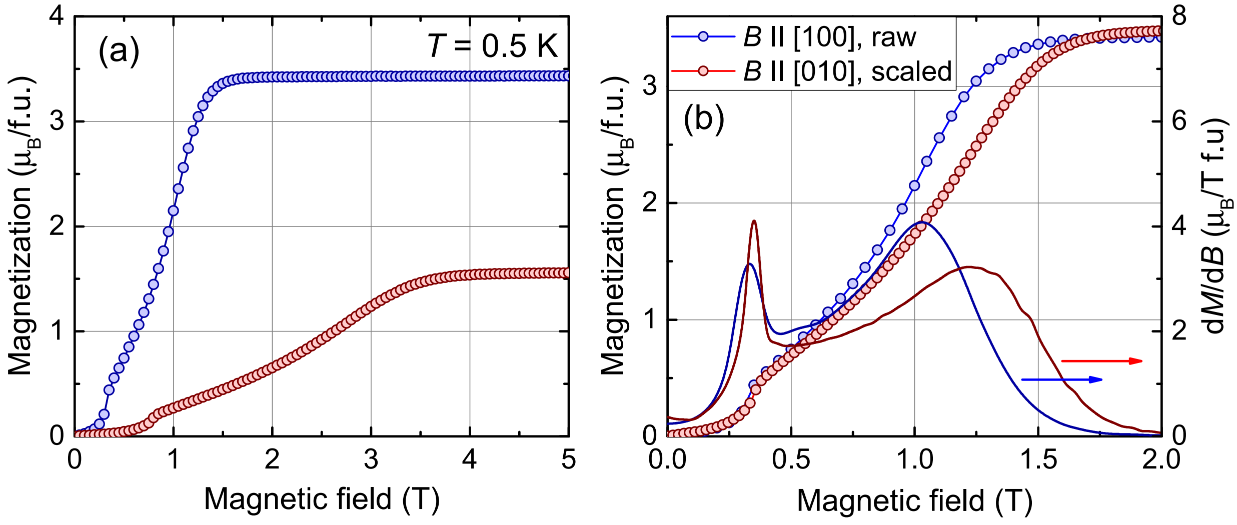

Supplementary Figure 1. Field-dependences of the magnetization of $YbAlO_3$ measured at $T = 0.5$ K along the a and b axes. Panel (a) shows the raw data; in panel (b) the b-axis magnetization is divided by tan(23.5°) and plotted as a function of $B \cdot \tan(23.5°)$. The solid lines in panel (b) show the field derivatives of the magnetization, dM/dB.

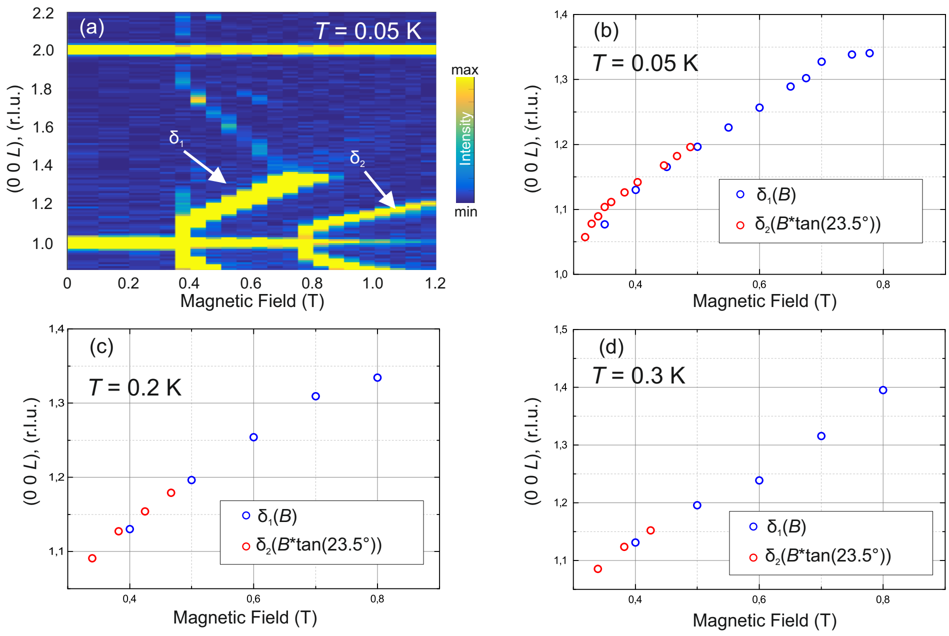

Supplementary Figure 2. (a) Magnetic field-dependence of the ENS intensity along the $(00L)$ direction measured on FLEXX at base temperature. White arrows show the positions of the δ_1 and δ_2 branches. (b-d) Field-dependence of $\delta_1(B)$ and $\delta_2(B \cdot \tan(23.5^\circ)$ at three different temperatures. Note that both branches collapse onto a single curve after taking into account the geometrical factor tan(23.5°). The positions of the peaks were obtained by fitting the raw ENS data with Gaussian functions.

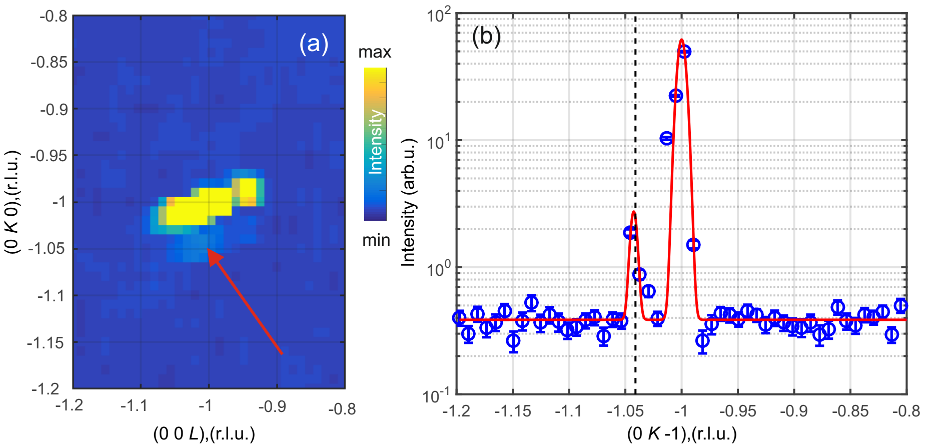

Supplementary Figure 3. Magnetic (0 -1 -1) Bragg peak measured on CNCS with $E_\mathrm{i} = 3.32$ meV at $T = 50$ mK. (a) Colour map of the ENS intensity in the $(0KL)$ scattering plane close to (0 -1 -1); the red arrow shows the position of the weak satellite. (b) Elastic cut along the (0 K -1) direction. Blue points show experimental data, red solid line its fit with two Gaussian functions plus a constant background; the vertical dotted line shows the position of the (-1 0 -1) reflection assuming the permutation $H \longleftrightarrow K$ described in the text.

To support this statement, in Supplementary Figure 1(a) we show the bulk magnetization measured at $T = 0.5$ K along the a and b axes. It is clear that both curves show qualitatively similar behaviour, although the saturation magnetization and critical field are somewhat different. In Supplementary Figure. 1(b) we plot $M_a(B)$ along with the scaled b-axis signal $\frac{M_b(B\tan(\alpha))}{\tan(\alpha)}$ and one can see very good agreement for the saturation field, as well as of the first critical field, between both curves. On the other hand, the second critical field is somewhat higher for the b-axis curve, most likely because the applied field produces an additional "staggered" field, which favours AFM order. These results indicate that the a- and b-axis responses are indeed qualitatively similar and mostly related by the simple trigonometric ratio $M_b/M_a = \tan(\alpha)$.

2. *Origin of the second splitting,* δ_2

$YbAlO_3$ exhibits unusual behaviour of the elastic scattering intensity above $B_{c1} = 0.32$ T. First, the (0 0 1) peak splits into $(0\ 0\ 1 \pm \delta_1)$, but a small part ($\simeq$ 5–7 %) of the intensity remains at the (0 0 1) position and does not change up to $B_{c1'} \approx 0.75$ T, where the second splitting, $(0\ 0\ 1 \pm \delta_2)$, takes place. Note that the $B_{c1'}$ coincides well with the expected first critical field for the case when the magnetic field is applied along the b-axis – $B_{c1}/\tan(\alpha) = 0.736$ T. As the next step, we plot $\delta_1(B)$ and $\delta_2(B \cdot \tan(\alpha))$ in Supplementary Figures 2(b-d) for three different temperatures and one can see a very accurate agreement of these three curves. This result provides strong evidence that our sample contains a small twin, which is oriented with its [010] axis parallel to the [100] axis of the primary crystallite.

3. *Diffraction*

When examining the diffraction map measured on CNCS at $T = 0.05$ K and $B = 0$ T, we found a weak satellite close to the magnetic (0 -1 -1) Bragg peak at slightly larger absolute K. Supplementary Figure 3(b) shows an elastic cut along the (0 K -1) direction of reciprocal space and one can see that the intensity of the satellites is around 3 % of the main peak. Moreover, assuming permutation of reciprocal coordinates, $H \longleftrightarrow K$, we calculated the expected position of the magnetic (-1 0 -1) reflection and it is shown by a vertical dotted line in Supplementary Figure 3(b), which again agrees well with the position of the observed reflection.

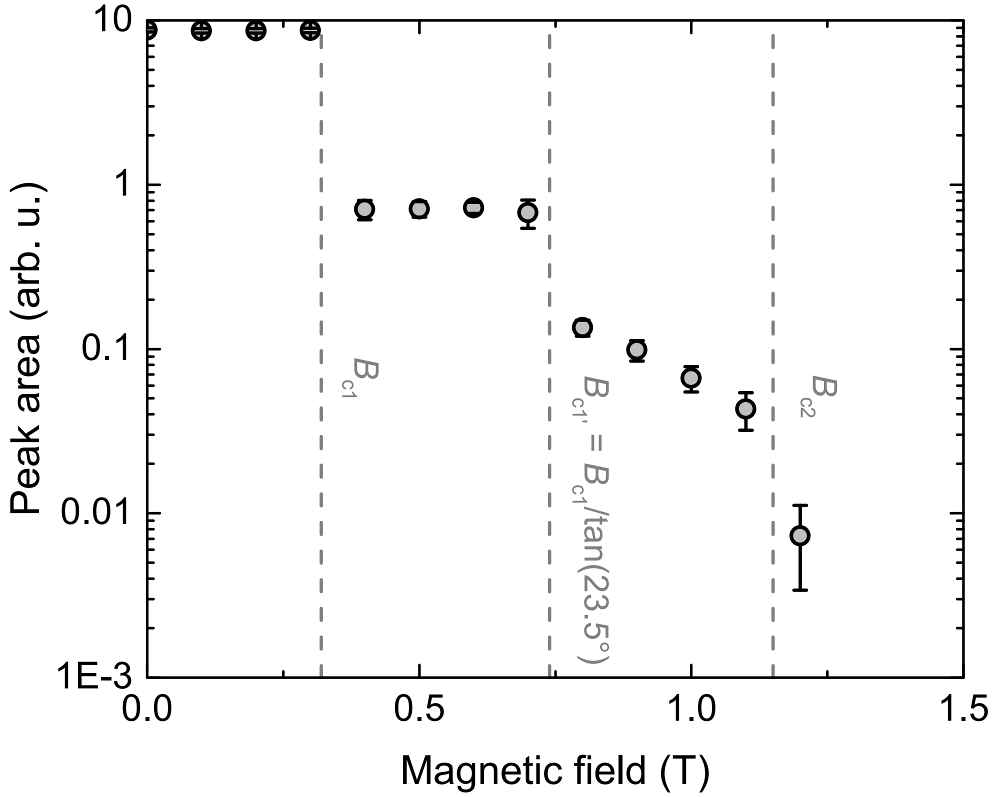

Supplementary Figure 4. Magnetic field-dependence of the (0 0 1) magnetic reflection measured using FLEXX at $T = 0.05$ K.

4. *Experimental consequences*

Above we provided experimental evidence that our sample contains crystallites with two orthogonal orientations. Now we discuss to what extent this circumstance limits the reliability of our data.

First of all, it is clear that below B_{c1} both parts of the sample are in the same AFM state and therefore contribute to the commensurate magnetic peak (0 0 1). In the field range between B_{c1} and $B_{c1'}$ the main sample is in the SDW phase with incommensurate order parameter $(0\ 0\ 1 \pm \delta_1)$, while the twin remains in the AFM phase, and manifests itself in the (0 0 1) magnetic reflection, which does not exhibit appreciable field-dependence between B_{c1} and $B_{c1'}$. Thus, the (0 0 1) peak between B_{c1} and $B_{c1'}$ is most likely related to scattering from the AFM ordered twin.

Above $B_{c1'}$ the twin enters the SDW phase and produces the second splitting, $(0\ 0\ 1 \pm \delta_2)$. Moreover, the intensity of the (0 0 1) peak shows a monotonic decrease with magnetic field and eventually disappears below the detection limit above 1.1 T, close to B_{c2} of the primary crystallite ($B \parallel [100]$). Therefore, above B_{c2} the peak at (0 0 1) may have two different origins: (i) it may be related to coexistence of the SDW and AFM phases within the twin, because the

AFM→SDW transition is known to be first-order; (ii) (0 0 1) can be the order parameter of the TAF phase, i.e. the S^{xx} and S^{yy} components of the structure factor of the main sample [7]. Accidentally, it turned out that $B_{c1'}$ for the twin is very close to the SDW-TAF phase boundary of the primary crystallite and because of the very close proximity of these field scales it is difficult to disentangle these two contributions unambiguously, and the real signal at (0 0 1) above $B_{c1'}$ most likely consists of both components. The field evolution of the (0 0 1) peak extracted from FLEXX measurements is summarized in Supplementary Figure 4.

To summarize, the presence of the twin does not affect our primary experimental results, which concern the behaviour of the incommensurate magnetic reflections within the SDW and TAF phases. Because of the relatively small volume of the twin, we do not see any manifestations of its presence in the inelastic channel. Moreover, we show that the second splitting, $(0\,0\,1\pm\delta_2)$, which we observed in our data is not an intrinsic feature of the spin-chain physics of $YbAlO_3$. Unfortunately, the twin strongly affects the intensity at the (0 0 1) reflection over the whole field range. For this reason we could not investigate $I_{(001)}(B)$, which is of special interest close to the QCP. To study the field-dependence of the TAF phase order parameter close to QCP, one has to either measure a twin-free sample or perform a careful polarization analysis on the currently available twinned samples. This said, it is worth noting that the growth of $YbAlO_3$ single crystals is extremely challenging even in the present twinned state, and the need for untwinned samples would set another challenge for chemistry.

S2. Neutron diffraction at different temperatures

Supplementary Figure 5 shows the ENS intensity as a function of magnetic field applied along the a axis, collected at different temperatures. At the base temperature, $YbAlO_3$ shows two incommensurate branches, $\delta_1(B)$ and $\delta_2(B)$. Moreover, the corresponding $2-2\delta_1(B)$ is also seen clearly in the colour map, Supplementary Figure 5(a). With increasing temperature, the incommensurate branches disappear at lower field, consistent with the phase diagram. The higher-order harmonics are also suppressed with increasing temperature.

S3. Extraction of the soft-mode position from the CNCS data

In order to find the **q**-point where the spectrum, $S^{zz}(q,\omega=0)$, reaches zero (the soft-mode point), we used the data measured on the CNCS. Because of the 1D behaviour of the magnetic dispersion, we integrated the data in both orthogonal directions over the whole available dataset. In order to avoid incoherent background, the integration energy window was set just

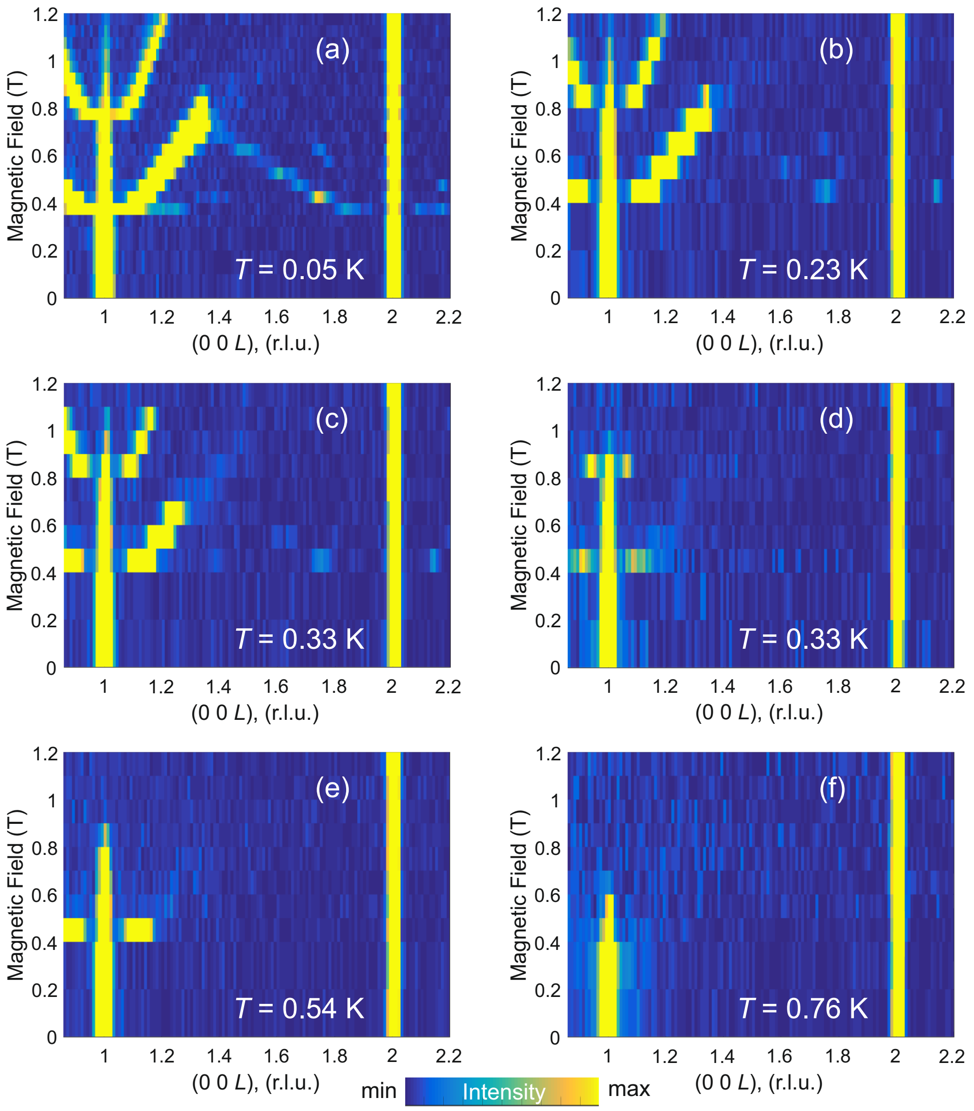

Supplementary Figure 5. Magnetic field-dependence of the ENS intensity along the $(0\ 0\ L)$ direction measured on FLEXX at different temperatures. Colour scales show the intensity of ENS – they are equivalent for each panel and oversaturated in order to highlight the weaker peaks.

above the elastic line, $E = [0.03\text{–}0.07]$ meV. Moreover, the subtraction of high-field dataset (which shows no magnetic signal) was applied to all the data.

Supplementary Figure 6 shows the resulting elastic cuts. The position of the soft mode was fitted with Gaussian functions and plotted as a function of magnetic field in Fig. 3(a) of the main text.

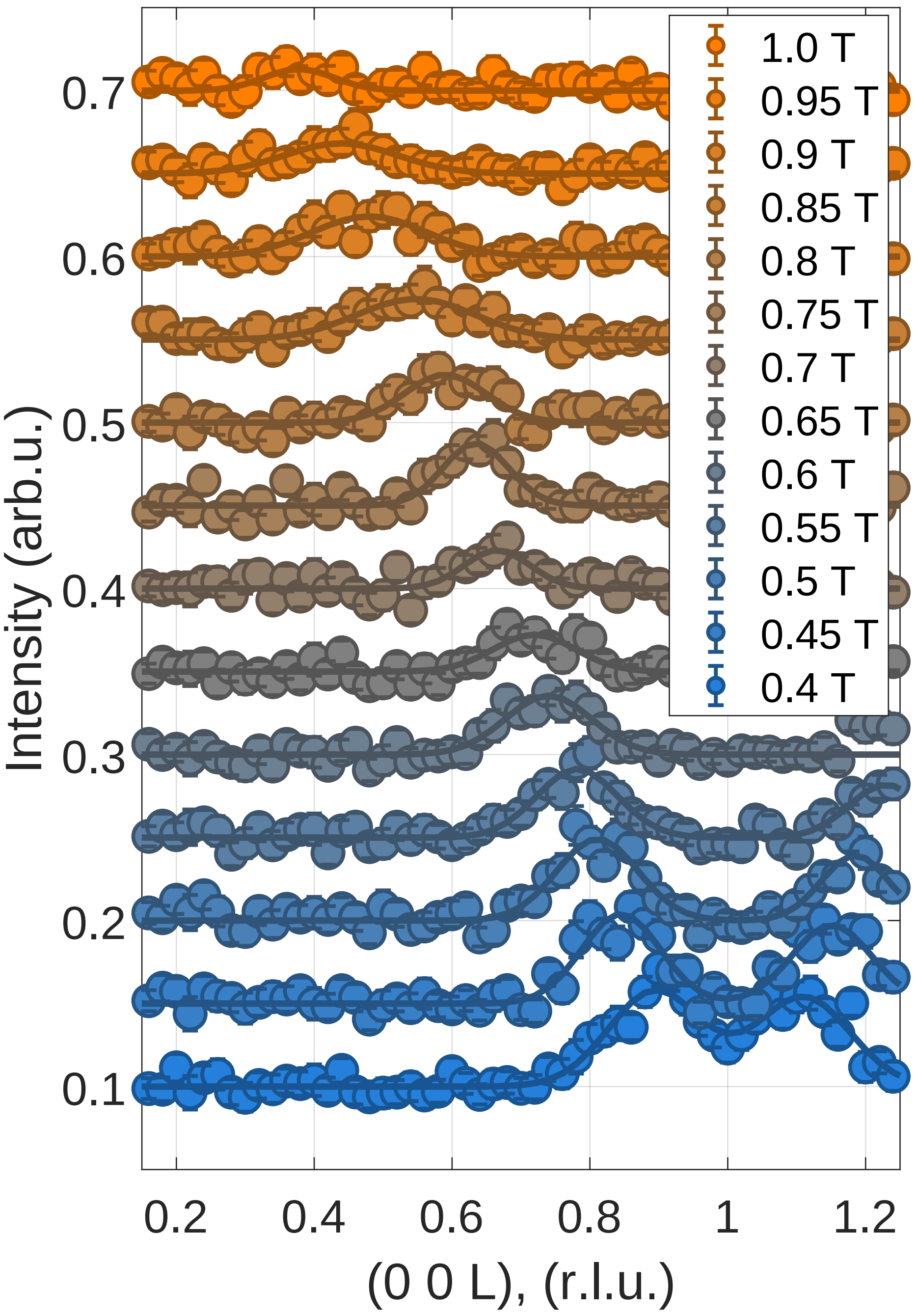

Supplementary Figure 6. ENS cuts along the $(0\ 0\ L)$ direction measured on the CNCS at $T = 0.05$ K. The curves were obtained by integrating the datasets close to the elastic line, $E = [0.04\text{–}0.07]$ meV; $K = 0 \pm 0.15$ (r.l.u.); $H = 0 \pm 0.1$ (r.l.u.). Solid lines show fits with Gaussian functions plus a constant background. The data are shifted vertically for ease of viewing.

S4. Bosonization

We first perform a Jordan-Wigner transformation to map the XXZ spin chain in Eq. (1) of the main text to interacting spinless fermions,

$$H = -\frac{J}{2}\sum_i (c_{i+1}^\dagger c_i + \text{h.c.}) + \Delta J \sum_i (c_{i+1}^\dagger c_{i+1} - \frac{1}{2})(c_i^\dagger c_i - \frac{1}{2}) - H_z \sum_i (c_i^\dagger c_i - \frac{1}{2}). \tag{S1}$$

The low-energy physics of this system is dominated by the left- and right-moving fermions close to the Fermi level. The single-particle operators of the right-moving (left-moving) fermions are defined as

$$\psi_{R/L}(x) \sim \sum_{k\sim \pm k_F} e^{ikx} c_k. \tag{S2}$$

We then follow the standard bosonization procedure [8, 9] by introducing the field $\phi(x,\tau)$ such that

$$S^z \simeq M + \frac{1}{\pi}\partial_x \phi + A\cos(2\phi - 2k_F x), \tag{S3}$$

where M is the magnetization of the spin chain and A is a nonuniversal constant. The Lagrangian of a Heisenberg spin chain in a magnetc field is then the Luttinger model,

$$\mathscr{L} = \frac{1}{\pi}\left[\frac{1}{u}(\partial_\tau \phi)^2 + u(\partial_x \phi)^2\right], \tag{S4}$$

where $u = \pi J/2$. This model describes a Tomonaga-Luttinger liquid (TLL) with dominant transverse spin correlations. Note that the intrachain Ising interaction is irrelevant at finite fields.

Now we consider two chains m and l. The Ising part of the interchain coupling induces a term

$$H_{ml} = J_{ab} S_m^z S_l^z \propto J_{ab}\cos(2\phi_m)\cos(2\phi_l). \tag{S5}$$

In the fermionic representation, this corresponds to Umklapp scattering processes between the left- and right-moving fermions of the form $\sim \psi_{L,m}^\dagger \psi_{R,m} \psi_{R,l}^\dagger \psi_{L,l}$, etc. Because of the different chain indices, Umklapp scattering processes transferring momentum $\sim 2k_F$ are a relevant perturbation to the Luttinger model in Eq. (S4), and stabilize the SDW order at $q = 2k_F = \pi(1 \pm 2m)$ for coupled spin chains.

Inside the SDW phase, one can treat the interchain coupling within a mean-field approximation, $H_{ml} \approx J_{ab}\langle S_m^z\rangle S_l^z$. This adds a term proportional to $J_{ab}\cos(2\phi)$ to the model of Eq. (S4), which is a relevant interaction acting as a backscattering, $J_{ab}(q)\psi_L^\dagger \psi_R$, with momentum transfer $q = 2k_F$ between the left- and right-moving fermions. This term leads to the folding of fermionic bands, as illustrated in Fig. 3(b) of the main text. Because $q = 2k_F$ is incommensurate, nq never equals to a reciprocal lattice vector, so there will be an infinite series of folded bands and,

correspondingly, the multiple scattering of the fermions with momentum $2nk_F$ is active (see Fig. 3(b)). Treating $J_{ab}\cos(2\phi)$ as a perturbation, it is easy to show that the cross-section of the nth scattering is proportional to J_{ab}^n.

S5. Details of the quantum Monte Carlo simulations

To determine the phase diagram and study the physical properties in the SDW phase, we consider an effective spin-1/2 model for $YbAlO_3$, which consists of a 3D array of weakly coupled Heisenberg spin chains. The Hamiltonian is

$$\mathcal{H} = J\sum_i \vec{S}_i \cdot \vec{S}_{i+c} - H_z \sum_i S_i^z + J_{ab} \sum_{i,\delta=\{a,b\}} \left[\varepsilon\left(S_i^x S_{i+\delta}^x + S_i^y S_{i+\delta}^y\right) + S_i^z S_{i+\delta}^z\right]. \quad \text{(S6)}$$

Here $\vec{S}_i = \{S_i^x, S_i^y, S_i^z\}$ is a spin-1/2 operator defined at site i. J and J_{ab} are respectively the intrachain and interchain exchange couplings between nearest-neighbour spins. H_z is the applied longitudinal magnetic field. Note that the longitudinal direction (z-axis) is different from the chain direction (c-axis) in $YbAlO_3$. ε denotes the spin anisotropy of the interchain coupling. In this work we take the intrachain interaction to be Heisenberg (isotropic in spin space) antiferromagnetic, with $J = 0.21$ meV. We also take a ferromagnetic interchain coupling with a strong Ising anisotropy, $J_{ab}/J = -0.2$, and anisotropy coefficient $\varepsilon = 0.25$, which produces phase boundaries that agree well with the experimental ones.

We examine the field-induced phase diagram of the model by performing numerically exact quantum Monte Carlo (QMC) simulations based on the stochastic series expansion (SSE) algorithm [10, 11]. In the simulations, the largest system size used is $20 \times 20 \times 128$ sites and the lowest temperature accessed is $T/J = 0.01$. We have calculated the normalized static longitudinal and transverse spin structure factors

$$S^{zz}(\mathbf{q}) = \frac{1}{N^2}\sum_{ij} e^{i\mathbf{q}\cdot(\mathbf{r}_i - \mathbf{r}_j)} \left\langle S_i^z S_j^z \right\rangle, \quad \text{(S7)}$$

$$S^{xy}(\mathbf{q}) = \frac{1}{2N^2}\sum_{ij} e^{i\mathbf{q}\cdot(\mathbf{r}_i - \mathbf{r}_j)} \left\langle S_i^x S_j^x + S_i^y S_j^y \right\rangle, \quad \text{(S8)}$$

where N refers to the total size of the system. The Néel AFM phase is characterized by a peak in the longitudinal structure factor, $S^{zz}(\mathbf{q})$, at wavevector $\mathbf{q} = (0, 0, \pi)$. In the longitudinal SDW phase, this peak splits into two parts, located at the incommensurate wavevectors $\mathbf{q} = (0, 0, \pi \pm \delta)$, respectively. The peaks diminish when the ground state changes to a TAF state, which is signaled by a peak in the transverse structure factor, $S^{xy}(\mathbf{q})$, at $\mathbf{q} = (0, 0, \pi)$. We also resolve satellite peaks located at $2\pi \pm 2\delta$, $\pi \pm 3\delta$, and $2\pi \pm 4\delta$, in the incommensurate SDW phase, shown in Fig. 3(d) of the main text, in agreement with the ENS measurements.

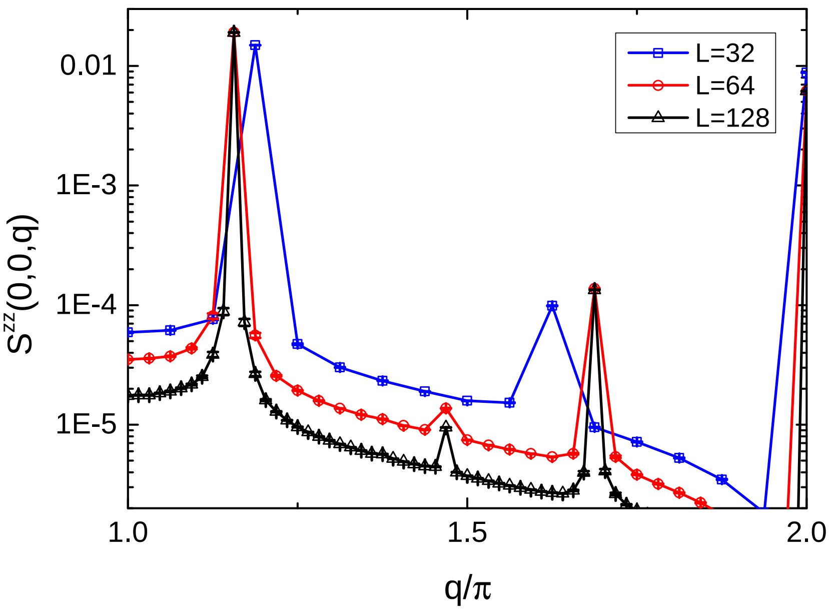

Supplementary Figure 7. Finite-size analysis of the longitudinal spin structure factor, $S^{zz}(0,0,q)$, computed at $h/J = 0.7$ in the QMC simulations. As the system size is increased in the chain direction, the peaks in $S^{zz}(\mathbf{q})$ converge to finite values and approach to incommensurate wavevector corresponding to the main and satellite peaks observed in neutron diffraction measurements.

Note that the QMC data are obtained for finite systems. In a finite system with chain length L, the allowed wavevectors are $2n\pi/L$, where n is an integer. In principle, the incommensurate ordering wavevector $q = 2k_F = \pi \pm \delta$ is not compatible with $2n\pi/L$ for any finite L. However, in the simulation, a certain integer number n can be obtained such that $2n\pi/L$ is a good approximation to the incommensurate q value, namely, n satisfies $2n\pi/L < q < 2(n+1)\pi/L$. The error in this approximation of the true wavevector in the simulation is then less than $2\pi/L$ and decreases with increasing system size. In Supplementary Figure 7 we show the finite-size analysis of the structure factor $S^{zz}(\mathbf{q})$, whose peaks clearly converge to finite values, which approach the incommensurate ordering wavevector of the longitudinal SDW phase. Our QMC data shown in the main text are obtained for the largest system with $L = 128$, where the finite-size effect is negligibly small on the scale of the figure.

S6. Origin of the $M_S/3$ plateau and DMRG calculations of two-leg spin ladder

In this section we consider the origin of the narrow $1/3$ magnetization plateau observed between $B = 0.67$ T and 0.76 T. First of all, it worth noting that our QMC calculations described in Sec. S5 do not reproduce the observed plateau. One possible reason is that the value of the

transverse component of the interchain coupling, which is parameterized by ε in Eq. (S6) is too large. For this reason the SDW is not stabilized up to $M = M_S/3$, as was observed in the experiment, but rather is suppressed in favour of the TAF phase at lower fields corresponding to $M \approx M_S/4$ (note that increasing of ε suppresses the SDW phase, as discussed in Ref. [12]). This choice was necessary because the QMC simulations become inefficient for smaller ε and therefore cannot provide reliable results in the limit $\varepsilon \to 0$. Moreover, the magnetization calculated within the SDW phase is rather noisy due to finite-size effects, which may cause a problem for resolving the experimental plateau, even if one could extend the SDW phase up to $M = M_S/3$ in the calculations.

As an alternative approach we used DMRG to study two Heisenberg spin chains coupled by an Ising interchain interaction. Previously, it was shown that this model captures the essential physics of $YbAlO_3$, although the magnetization of this model does not show the plateau [13]. However, it is known that the magnetization plateau emerges for a spin-1/2 system only when the quantity $n(1-m)/2$ is an integer, where n is the number of sites included in the magnetic unit cell of the ground state [14]. Thus, the magnetic period of the 1/3 plateau state must be $n = 3$, where the system is in a translational-symmetry-breaking state with a magnetic unit cell consisting of one polarized spin, while the other two spins form a valence bond. In $YbAlO_3$, we assumed that the translational symmetry is broken along the chain direction. In fact, this is consistent with the peak position extracted from the INS spectra around the 1/3 plateau, $q_L \sim 2\pi/3$. This kind of symmetry breaking can be achieved in model calculations by adding a next-nearest-neighbour (NNN) AFM interaction, J', in the chain.

To obtain a fit of the experimental magnetization curve, we made a grand canonical DMRG analysis employing the sine-square deformation function [15, 16]. This method gives a numerically exact and unbiased magnetization curve in the thermodynamic limit, and enables one to detect very narrow plateaux [17]. In brief, the method divides the finite-size cluster smoothly into a centre part and the edges, and the central part reproduces the continuous bulk response by using the nearly zero-energy edge state as a buffer. At a fixed system size and shape, we introduce the modulation of the energy scale by an externally imposed function, $f(r)$, which smoothly deforms the Hamiltonian from the maximum at the centre of the system ($r = 0$) to zero energy at the open cluster edges ($r = R$). After we obtain the eigenfunctions of the deformed Hamiltonian, we evaluate the magnetization, $\langle S^z(r)\rangle$. While the total magnetization, $M_{\rm tot} = \sum\langle S^z(r)\rangle$, of the deformed Hamiltonian is a conserved and externally imposed quantity, the expectation value of the local magnetic density, $\langle S^z(r)\rangle$, is no longer equal to $M_{\rm tot}/N$, but has a particular r-dependence: it takes nearly a uniform value at the centre (although oscillating slightly, the mean values are uniform) and then often takes a peak or valley at the edges. This

is because the system optimizes the wave function to realize a centre value $\langle S^z(r \sim 0)\rangle$ near the thermodynamic limit, M, at any given magnetic field. The magnetization, $M_{\mathrm{tot}}/N - M$, is provided by the localized edge states of the cluster, which have an energy of measure zero as $f(r = R) \sim 0$. These edges serve as a grand canonical bath. The reason why this mechanism works is discussed in detail in Ref. [16] in the context of real-space energy renormalization,

In the present analysis, the original Hamiltonian consisting of local terms $\mathscr{H}_i$, defined as in Ref. [15], is deformed as

$$\mathscr{H}_{\mathrm{deform}} = \sum_{i=1}^{L} \mathscr{H}_i f(r_i), \tag{S9}$$

where $f(r)$ is an externally imposed function, which varies smoothly from a maximum at the centre of the cluster $[i = (L+1)/2]$ to zero at the edges of the cluster. For such a function, we typically adopt then sine-square deformation (SSD) function, which provides a smooth boundary condition. For the 1D system, the SSD function is given as

$$f_{\mathrm{SSD}}(i) = \sin^2\left(\frac{\pi}{L}(i - \frac{1}{2})\right) \tag{S10}$$

with either $i = 1, \cdots, L$ for on-site terms or $i = 3/2, \cdots, L - 1/2$ for interaction terms between sites i and $i + 1$.

We used DMRG as a solver for the deformed Hamiltonian in the grand canonical analysis. Because the interchain couplings form a bipartite square lattice in the a-b plane and no exchange processes are allowed between the chains, the extension of the cluster in the a-b plane can be reduced to a two-chain problem. Hence, we studied two isotropic Heisenberg chains coupled by a ferromagnetic Ising interaction. We note that, in order to count the strength of the interchain coupling consistently in comparison with the material, the interchain Ising coupling should be replaced by $\widetilde{J}_{\mathrm{ic}} = 4J_{\mathrm{ic}}/N_{\mathrm{c}}$, where N_{c} is the number of neighbouring chains, i.e., $N_{\mathrm{c}} = 1$ for two chains. We studied clusters with length $L \times 2 = 120 \times 2$ and kept up to $m = 2000$ density-matrix eigenstates in the renormalization procedure. In this way, the maximum truncation error, i.e., the discarded weight, was less than 1×10^{-11}.

The fitting result is shown in Supplementary Figure 8. The best fit is obtained by setting $J = 3.75$ K, $J' = 0.75$ K, and $J_{\mathrm{ic}} = -3.56$ K ($J'/J \simeq 0.2$ and $|\widetilde{J}_{\mathrm{ic}}/J| \simeq 0.95$). These values of parameters are comparable to the ones used in the QMC simulation. As seen in the inset of Supplementary Figure 8, the DMRG curve reproduces the 1/3 plateau rather well, although the experimental plateau is partially destroyed by thermal fluctuations. Further, we note that the divergent behaviour of M around the saturation field, a signature of strong one-dimensionality of the Tomonaga-Luttinger liquid, is rounded at $T = 0.05$ K.

We remark that the ratio $J'/J \sim 0.2$ does not in fact fulfill the typical emergence condition for the 1/3 plateau, $J'/J \geq 0.571$, in the SU(2) J-J' Heisenberg chain [18]. This anomaly can

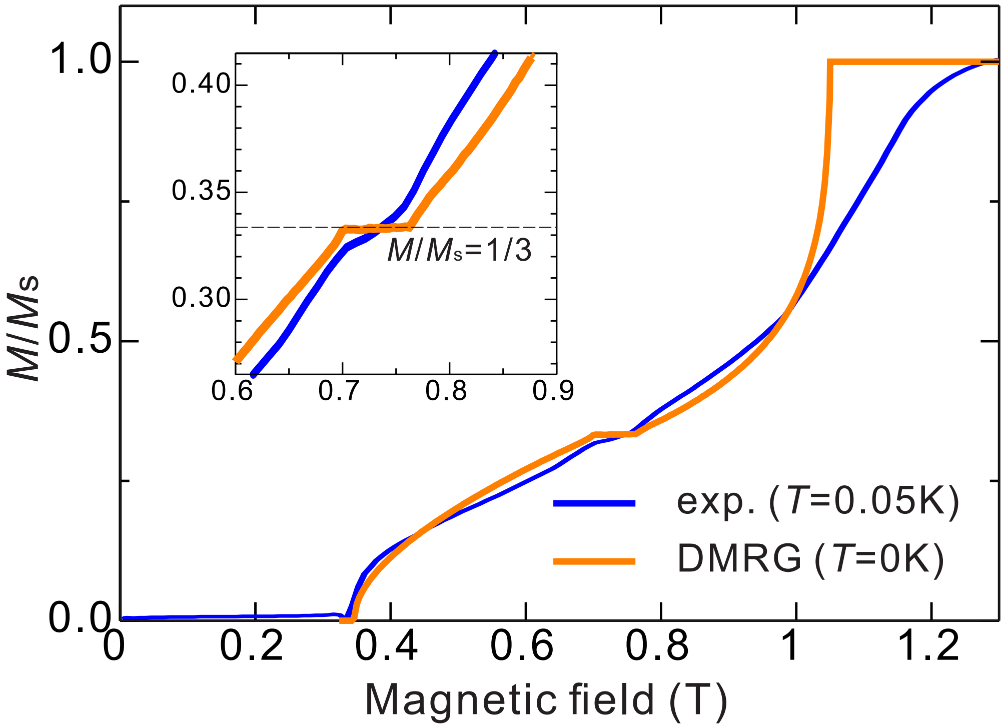

Supplementary Figure 8. Fitting of the experimental magnetization curve for $B \parallel a$ by DMRG with coupled J-J' chains, where the experimental magnetization is normalized by the a-axis g-factor of $YbAlO_3$, $g_a = 7.6$. Inset: enlarged figure around the 1/3 plateau.

be explained by taking account of the peculiar lattice structure of $YbAlO_3$. In $YbAlO_3$, either Ising anisotropy or a staggered field is effectively induced in each chain due to the Ising-type interchain coupling [6, 13]. In such cases, the 1/3 plateau can emerge with a relatively small J'/J [18]. This small J'/J also provides an additional contribution acting to preserve the commensurate AFM ordering at low fields, because the NNN AFM interaction significantly increases the incommensurate fluctuations along the chain even at zero field. Thus the presence of a weak intrachain next-neighbour frustrating interaction, J', provides one explaination for the observed plateau. However, theoretical investigations of a more realistic spin model, which takes into account aspects such as long-range dipolar interactions, should be performed to give a final answer on the nature of the observed plateau.

---

[1] O. Buryy, Ya. Zhydachevskii, L. Vasylechko, D. Sugak, N. Martynyuk, S. Ubizskii, and K. D. Becker, "Thermal changes of the crystal structure and the influence of thermo-chemical annealing on the optical properties of $YbAlO_3$ crystals," J. Phys. Condens. Matter **22**, 055902 (2010).

[2] S. B. Ubizskii, L. O. Vasylechko, D. I. Savytskii, A. O. Matkovskii, and I.M. Syvorotka, "The crystal structure and twinning of neodymium gallium perovskite single crystals," Supercond. Sci. Tech. **7**,

766 (1994).

[3] D. I. Savytskii, L. O. Vasylechko, A. O. Matkovskii, I. M. Solskii, A. Suchocki, D. Yu. Sugak, and F. Wallrafen, "Growth and properties of $YAlO_3$:Nd single crystals," J. Cryst. Growth **209**, 874–882 (2000).

[4] D. Savytskii, A. Senyshyn, A. Matkovskii, L. Vasylechko, K. Wieteska, W. Wierzchowski, T. Lukasiewicz, and U. Bismayer, "White beam synchrotron X-ray topography studies of twinning in $GdFeO_3$-type perovskite crystals," Z. Kristallogr. Cryst. Mater. **218**, 17–25 (2003).

[5] L.S. Wu, S.E. Nikitin, M. Brando, L. Vasylechko, G. Ehlers, M. Frontzek, A. T. Savici, G. Sala, A. D. Christianson, M. D. Lumsden, and A. Podlesnyak, "Antiferromagnetic ordering and dipolar interactions of $YbAlO_3$," Phys. Rev. B **99**, 195117 (2019).

[6] L.S. Wu, S.E. Nikitin, Z. Wang, W. Zhu, C.D. Batista, A.M. Tsvelik, A.M. Samarakoon, D.A. Tennant, M. Brando, L. Vasylechko, M. Frontzek, A.T. Savici, G. Sala, G. Ehlers, A.D. Christianson, M.D. Lumsden, and A. Podlesnyak, "Tomonaga-Luttinger liquid behavior and spinon confinement in $YbAlO_3$," Nat. Commun. **10**, 698 (2019).

[7] Yuchen Fan, Jiahao Yang, Weiqiang Yu, Jianda Wu, and Rong Yu, "Phase diagram and quantum criticality of Heisenberg spin chains with Ising anisotropic interchain couplings," Phys. Rev. Research **2**, 013345 (2020).

[8] T. Giamarchi, "Quantum Physics on One Dimension," (Oxford University Press, 2003) ISBN: 0-19-852500-1.

[9] K. Okunishi and T. Suzuki, "Field-induced incommensurate order for the quasi-one-dimensional XXZ model in a magnetic field," Phys. Rev. B **76**, 224411 (2007).

[10] O. F. Syljuåsen and A. W. Sandvik, "Quantum Monte Carlo with directed loops," Phys. Rev. E **66**, 046701 (2002).

[11] F. Alet, S. Wessel, and Matthias M. Troyer, "Generalized directed loop method for quantum Monte Carlo simulations," Phys. Rev. E **71**, 036706 (2005).

[12] Yuchen Fan and Rong Yu, "Role of the spin anisotropy of the interchain interaction in weakly coupled antiferromagnetic heisenberg chains," Chinese Physics B **29**, 057505 (2020).

[13] C. E. Agrapidis, J. van den Brink, and S. Nishimoto, "Field-induced incommensurate ordering in Heisenberg chains coupled by Ising interaction: Model for ytterbium aluminum perovskite $YbAlO_3$," Phys. Rev. B **99**, 224423 (2019).

[14] Masaki Oshikawa, Masanori Yamanaka, and Ian Affleck, "Magnetization Plateaus in Spin Chains: "Haldane Gap" for Half-Integer Spins," Phys. Rev. Lett. **78**, 1984–1987 (1997).

[15] Chisa Hotta and Naokazu Shibata, "Grand canonical finite-size numerical approaches: A route to measuring bulk properties in an applied field," Phys. Rev. B **86**, 041108 (2012).

[16] Chisa Hotta, Satoshi Nishimoto, and Naokazu Shibata, "Grand canonical finite size numerical approaches in one and two dimensions: Real space energy renormalization and edge state generation," Phys. Rev. B **87**, 115128 (2013).

[17] Satoshi Nishimoto, Naokazu Shibata, and Chisa Hotta, "Controlling frustrated liquids and solids with an applied field in a kagome Heisenberg antiferromagnet," Nature communications **4**, 1–6 (2013).

[18] Kouichi Okunishi and Takashi Tonegawa, "Fractional S^z excitation and its bound state around the 1/3 plateau of the $S = 1/2$ Ising-like zigzag XXZ chain," Phys. Rev. B **68**, 224422 (2003).
